# Supramolecular Encapsulation of a Neurotransmitter Serotonin by Cucurbit[7]uril

**DOI:** 10.3389/fchem.2020.582757

**Published:** 2020-10-23

**Authors:** Falguni Chandra, Tanoy Dutta, Apurba L. Koner

**Affiliations:** Bionanotechnology Laboratory, Department of Chemistry, Indian Institute of Science Education and Research Bhopal, Bhopal, India

**Keywords:** supramolecular encapsulation, cucurbit[7]uril, neurotransmitter, serotonin, complexation induced NMR shift, competitive assay, drug delivery

## Abstract

pH-dependent host-guest complexation of a monoamine neurotransmitter, Serotonin, with cucurbit[7]uril has been thoroughly investigated. The binding phenomena were explored using steady-state and time-resolved fluorescence spectroscopy at different pH values. At lower pH, i.e., protonated Serotonin, the binding affinity with cucurbit[7]uril was significantly higher compared to higher pH. Furthermore, detailed NMR titration experiments depicted the solution structure of the host-guest complex through the complexation induced chemical shift values. A competitive binding assay with cesium ions at pD 2.8 was subsequently performed for the further manifestation of the binding. Finally, the molecular docking studies provided well-documented proof of the 1:1 inclusion complex and the geometry of the complex. We believe that understanding from such studies can be important for pH-controlled delivery of serotonin for biological applications.

## Introduction

**Figure 1:**
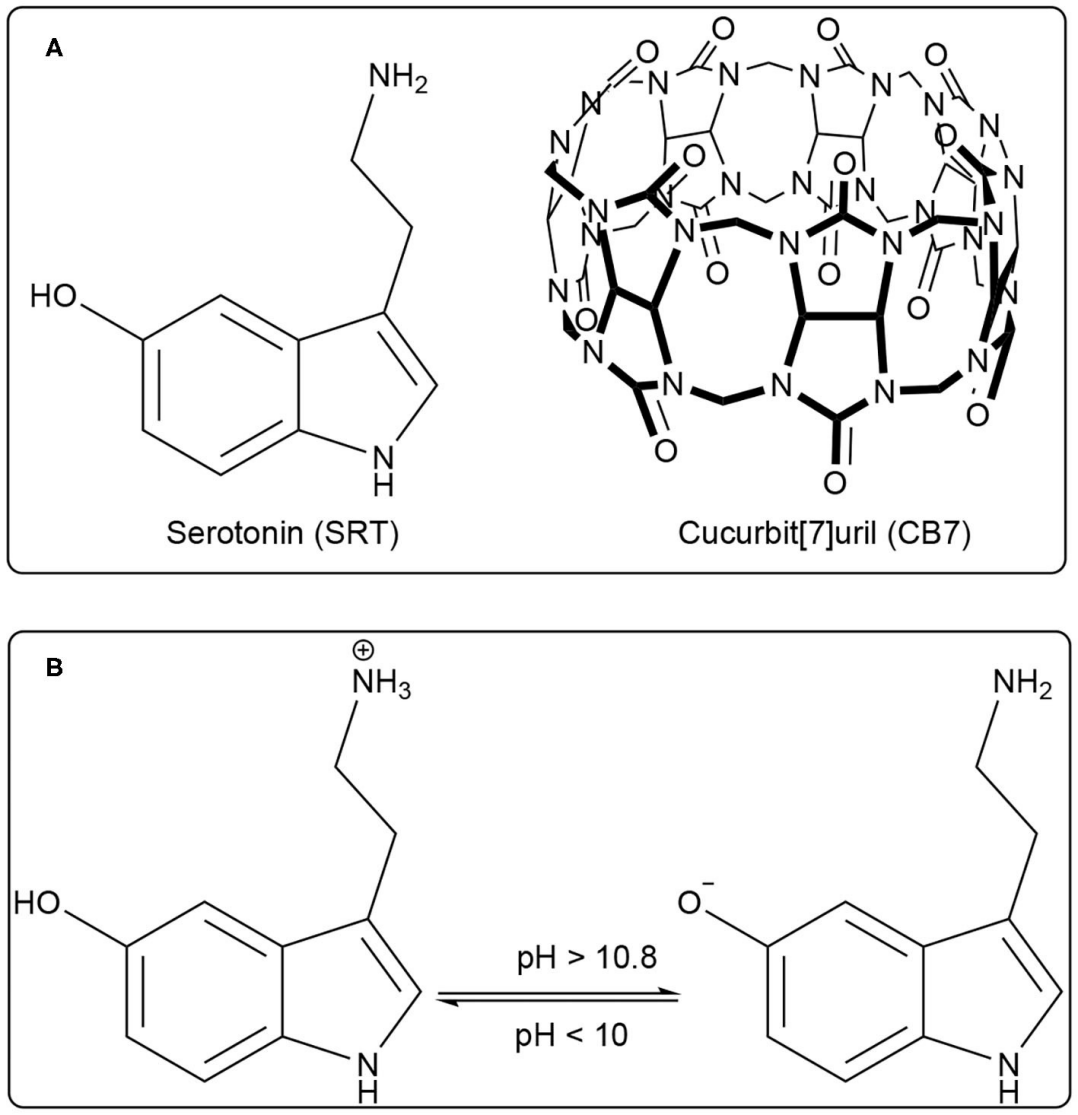
**(A)** Chemical structure of Serotonin (SRT, guest) and Cucurbit[7]uril (CB7, host), **(B)** Prototropic equilibrium of SRT with different pH.

Serotonin (5-hydroxytryptamine, SRT, for structure see Figure 1A) is a naturally occurring tryptamine derivative and biogenic amine acts as a neurotransmitter for both central as well as the peripheral nervous system. It was discovered by an Italian pharmacologist Vittorio Erspamer in 1952, identifying enteramine as 5-hydroxytryptamine (Erspamer and Asero, 1952). Later, SRT was synthesized by a second tryptophan hydroxylase isoform (Walther et al., 2003). It is exclusively found in all types of mammals including the human gastrointestinal tract, blood platelets, and nervous system. Approximately 80–90% of the total SRT is located in the enterochromaffin cells in the gut of humans and the remaining 10–20% is synthesized in serotonergic neurons in the Central Nervous System (CNS) and blood platelets (Mawe and Hoffman, 2013; Lv and Liu, 2017). SRT is widely distributed in the brain (Carhart-Harris and Nutt, 2017) and controls a variety of physiological and behavioral processes e.g., sleep, addiction, sexual activity, aggression, locomotion, anxiety, cognition, and food intake (Veenstra-Vanderweele et al., 2000; Berger et al., 2009). Especially, the food intake takes a significant dip due to activated serotonergic activity causing short-term food deprivation (Johnston and Glanville, 1992; Ruibal et al., 2002). The unwanted disruption of SRT balance in body systems may cause mental disorders such as Alzheimer's disease, infantile autism, schizophrenia, and depression (Dubovsky and Thomas, 1995; Voet and Voet, 2006). SRT is intrinsically fluorescent in the living system and it emits around 335 nm (Hernandez-Mendoza et al., 2020). Kishi and co-workers reported that the SRT shows different fluorescence properties at various pH values (Kishi et al., 1977). Chattopadhyay and co-workers reported a detailed characterization of its photophysical properties upon modulation by ionization and polarity of the medium (Chattopadhyay et al., 1996). Various literature reported that the SRT has two acid dissociation constant (p*K*_a_) value, i.e., one is 9.97 for an aliphatic amino group and 10.73 (see Figure 1B) for an aromatic hydroxyl moiety (Chattopadhyay et al., 1996; Pratuangdejkul et al., 2006). Ouyang and Vogel studied the interaction of SRT with metal-binding protein calmodulin using UV-Vis., fluorescence, and NMR spectroscopic techniques (Hui and Vogel, 1998). Considering the biological importance of SRT, its delivery using a supramolecular approach will be highly desirable for the therapies associated with SRT syndrome.

Water-soluble macrocyclic host molecules are very important for encapsulating polar and non-polar guest molecules with an offering of a hydrophobic cavity. Macrocycle molecules can have a quite rigid and well-defined cavity which can strongly encapsulate hydrophobic drug molecules with reasonable binding constant. The macrocyclic host molecules build a supramolecular host-guest system which provides an opportunity to study the nature of intermolecular interactions (Dsouza et al., 2011). Interestingly, the host-guest complexation is untangling of the dynamic nature of the formation of simple supramolecular assembly. The interaction of small organic molecules with a macrocyclic water-soluble host molecule is the prototype example of supramolecular interaction. Calixarenes, cyclodextrins, charged-cyclophane, and crown ethers, the popular drug-delivery carriers, are water-soluble macrocyclic host molecules and they have variable cavity size which is of nano-dimension with changing the monomer unit (Ghosh and Nau, 2012; Zhang et al., 2020). In comparison, a new class of water-soluble macrocyclic host molecule, cucurbit[n]urils (CBn, *n* = 5–10) are composed with “*n*” glycoluril units bridged by methylene groups (Nau et al., 2011). CBn has two symmetrical portals made of “*n*” carbonyl groups. CB7 has seven carbonyl groups presented in both the portals (Figure 1A). Both the portals of CBn are capable of binding positively charged molecules or ions. The cationic guest molecules comprising of a hydrophobic part are encapsulated in the hydrophobic nano-cavity of CBn driven by ion-dipole interaction, hydrogen bonding with carbonyl portals (Mondal et al., 2015; Ahmed et al., 2016; Yin and Wang, 2018). Such encapsulation causes a significant shift in the acid-dissociation constant and can effectively cause enhanced solubility of the guest, controlled release for drug delivery applications, and sensing using electronic spectroscopy for optically active molecules (Saleh et al., 2008, 2011, 2016; Wang et al., 2009; Koner et al., 2011; Ghosh and Nau, 2012; Lazar et al., 2016; Mallick et al., 2016; Kuok et al., 2017; Yin et al., 2017; Das et al., 2019; Wu et al., 2019) The formation of ternary complex with guest molecues and CBn is frequently obtained and formation of such the complexes have produced multifaceted applications (Bhasikuttan et al., 2007; Choudhury et al., 2010; Barooah et al., 2017). In last decade, new derivatives of CB are getting significant popularity and showed immense potential for various applications (Dsouza et al., 2011; Cong et al., 2016; Gao et al., 2017; Liu et al., 2019a,b).

Of late, our group reported the detailed photophysical properties of anti-malarial drug Quinine and β-carboline-based drug Norharmane upon binding with CB7 (Mallick et al., 2016; Chandra et al., 2018). The encapsulation resulted in greater solubility and excited state p*K*_a_ shift in both the cases which impacts reduced phototoxicity, improved bioavailability of the drug molecules. In view of further advancement in this field, we took interest in serotonin (SRT) which has manifold roles in our physiological system. We have studied the complexation of SRT by CB7 using UV-Vis. and fluorescence spectroscopy, time-resolved anisotropy, NMR spectroscopy, and molecular docking studies. A significant difference in pH-dependent binding affinity of SRT was observed upon CB7 encapsulation. As the complexation-induced chemical shifts helped to understand the orientation of SRT inside the CB7 cavity, the docking studies provided with the complex geometry along with the stoichiometry.

## Materials and Physical Methods

Serotonin (5-hydroxytryptamine) was purchased from Alfa Aesar (USA). HCl and NaOH were purchased from SDFCL, India) and Rankem, India, respectively. The pH of the solutions was adjusted using dilute HCl and NaOH solution. D_2_O, DCl, and NaOD were purchased from Sigma Aldrich, USA. All chemicals were used as received without any further purification. CB7 was synthesized and purified according to the previous report (Marquez et al., 2004) and characterized by ^1^H NMR spectroscopy and mass spectrometry. The experiments in water were carried out using Milli-Q grade water using Milli-Q water purification set up from Merck (USA) with resistivity 18.2 MΩ·cm at 298 K.

### Steady-State Absorption and Fluorescence Spectroscopy Experiments

All steady-state absorption and fluorescence measurements were carried out using Cary 5000 UV-Vis. spectrophotometer (Agilent Technologies) and HORIBA Jobin Yvon Fluorolog 3 instrument, respectively. Fluorescence spectra were recorded from 290 to 450 nm by exciting at 280 nm. All measurements were performed with a 1 cm path length quartz cuvette keeping both excitation and emission slit width 2 nm. All the experiments were carried out using a 10 μM SRT at room temperature (298 K). A concentrated stock solution of 1 mM SRT was prepared in Milli-Q water and diluted accordingly using Milli-Q water for absorption and fluorescence spectroscopic measurements. A dilute solution of the dye was taken for all the measurements to keep the absorption value low to avoid the inner filter effect. pH titrations by UV-Vis and fluorescence were performed in Milli-Q water and the pH/pD of the solution was adjusted by adding a minimal amount of concentrated HCl/DCl and NaOH/NaOD solution in water to avoid any dilution effect. The pH/pD was also measured at the end of the titration to test the pH/pD stability during titration.

### Time-Resolved Experiments

Time-resolved fluorescence and anisotropy decay measurement were performed using a Hamamatsu MCP photomultiplier (R-3809U-50). The time-correlated single-photon counting (TCSPC) setup consists of an Ortec 9327 pico-timing amplifier and using a pulsed Diode laser (λ_ex_ = 280 nm) with fwhm ~143 ps with a setup target 10,000 counts. The instrument response function (IRF) was collected using a dilute suspension of Ludox (colloidal silica, purchased from Sigma Aldrich). The emission and excitation polarizer was set at a magic angle (54.75°) to each other. The mono and bi-exponential fitting functions were employed by iterative deconvolution method using software DAS v6.2. The quality of the fitted data was judged from the reduced chi-squared value (χ^2^), calculated using the IBH software provided with the instrument. Following is the type of fitting function used.

$$\begin{array}{l} \frac{\text{I}\left(\text{t}\right)}{\text{I}\left(0\right)}=\sum {\text{a}}_{\text{i}}\text{e}\text{x}p\left(\frac{-\text{t}}{\tau_{\text{i}}}\right) \end{array}$$

### NMR Titration

^1^H NMR spectra were recorded using Bruker Avance III 400 MHz NMR spectrometer. Chemical shifts (δ) values are reported in ppm. All NMR titrations were performed in D_2_O at 298 K and the pD value of the solution was adjusted using NaOD and DCl. Dilute solutions of NaOD and DCl were prepared by diluting their concentrated stock in D_2_O. For NMR titration, the concentration of SRT was kept fixed to 0.5 mM in D_2_O and a concentrated stock solution of CB7 with same pD value was added accordingly to get the desired concentration. All NMR spectra from a binding titration were obtained on the same day and the data was collected immediately after making the respective solution.

### Molecular Docking

Molecular docking studies of SRT•CB7 inclusion complex was performed using the PatchDock server (Duhovny et al., 2002; Schneidman-Duhovny et al., 2005). The input files were optimized initially and uploaded to the server in PDB format. PatchDock uses different sets of parameters for the complex type field settings to automatically determine complementarity determining regions (CDR) of the host/receptor molecule. In the final stage of clustering, RMSD (root mean square deviation) clustering is performed to discard the superfluous modeled structures. The output is based on the geometric score, interface area size, and atomic contact energies of the docked structures.

## Results and Discussions

### pH-Dependent UV-Vis. and Fluorescence Measurements

**Figure 2:**
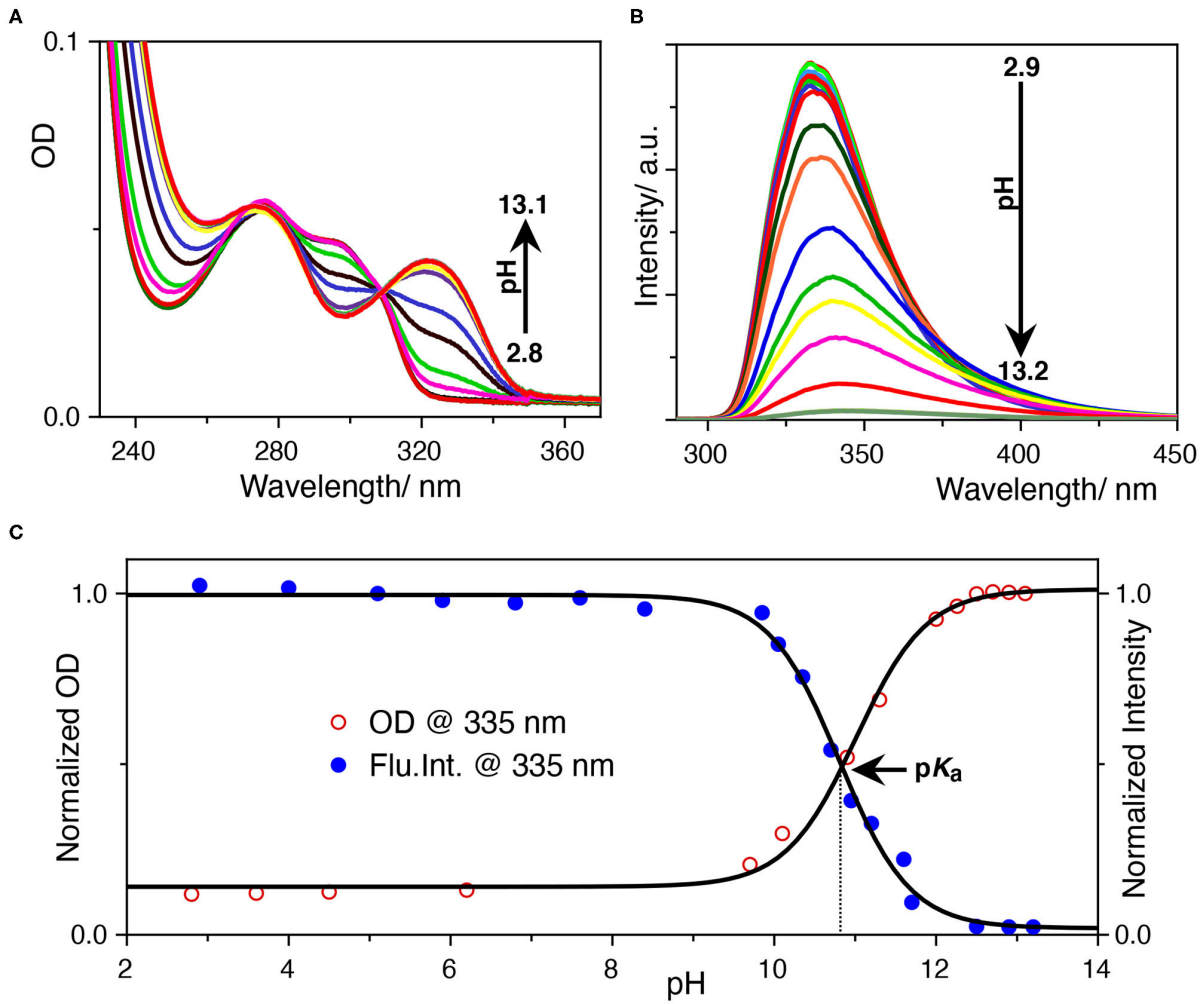
**(A)** UV-Vis.-based pH titration, the 333 nm absorption peak increases with pH, **(B)** fluorescence-based pH titration, the fluorescence peak at 335 nm decreases with pH, **(C)** determination of the p*K*_a_ value from UV-Vis. and fluorescence titration. The normalized optical density at 333 nm and the fluorescence intensity at 335 nm was plotted against the pH of the solution. The data points were fitted with a sigmoidal fitting function using Origin pro software to obtained the p*K*_a_ value.

The photophysics of SRT is sensitive to the pH of the solution. In acidic pH, it exhibits two absorption maxima at 277 and 297 nm while increasing the pH, 297 nm band diminishes and gives rise to another one with a maxima at 325 nm (Figure 2A). The isosbestic point at 309 nm signifies the conversion between neutral and protonated species. The origin of the red-shifted band at 325 nm indicates an increase in the conjugation of the chromophore unit. This could only be because of the deprotonation of the hydroxyl group present at the 5-position of the indole ring. After deprotonation of the phenolic -OH group, the negative charge of the phenolate ion has participated in the ring resonance and increased the conjugation length of the indole ring. Thereafter, we have performed a sigmoidal fitting of the optical density data measured at 335 nm obtained from UV-Vis. titration. We have obtained a p*K*_a_ value of 10.8 for the phenolic -OH group (Figure 2C). To understand the effect of pH on the emission properties of SRT, the pH-dependent fluorescence spectra were recorded upon excitation at 280 nm at different pH. It was observed that the intensity became negligible with the increasing pH. This phenomenon can be attributed to the lower absorbance value of the ionized SRT at the excitation wavelength, which leads to the gradual decrease in the intensity above physiological pH. Similar to the absorption, we have also obtained a p*K*_a_ value of 10.8 from the fluorescence titration (Figures 2B,C). It is reported that the p*K*_a_ value of the primary amine of SRT is 9.9 (Chattopadhyay et al., 1996; Pratuangdejkul et al., 2006). So, below this pH value, the primary amine group is also protonated and bears a positive charge. The p*K*_a_ value obtained from the pH titration using absorption and emission spectroscopy are identical. This indicates that there is no change in the p*K*_a_ value upon electronic excitation.

### Binding Studies and Time-Resolved Anisotropic Behavior of the Complex From Fluorescence

**Figure 3:**
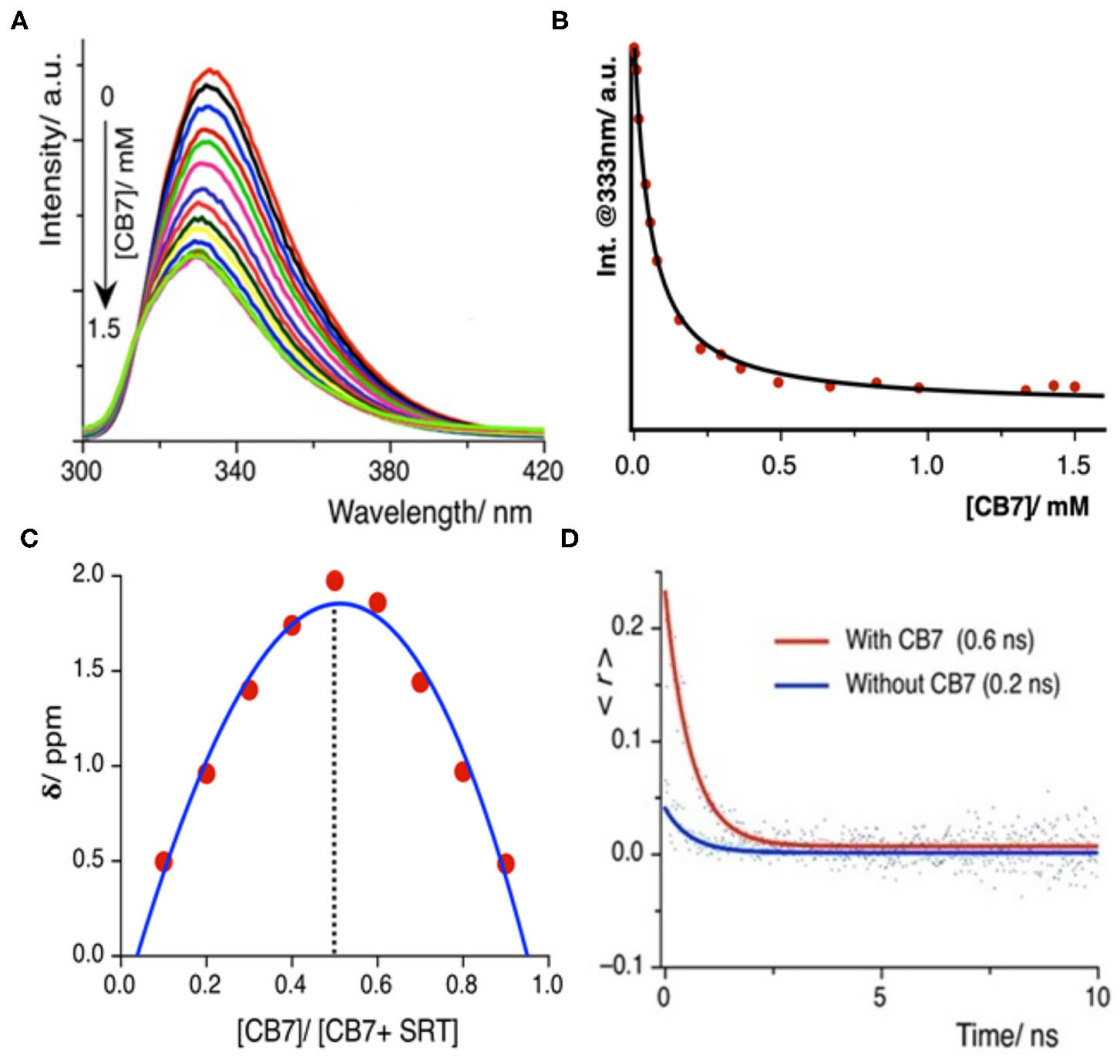
**(A)** Fluorescence titration of SRT with CB7 at pH 3.0; intensity gradually decreases upon encapsulation, **(B)** the fitted plot using a 1:1 binding equation yields a binding constant value (23,000 ± 500) M^−1^ **(C)** Job's plot using the change in chemical shift against the relative concentration of CB7 shows 1:1 binding stoichiometry, **(D)** time-resolved anisotropy decay plot of SRT in presence and absence of CB7 at pH 3.

Once we explored the pH-sensitivity in the ground and excited-state photophysical properties of SRT, the complexation with CB7 was to be well-understood. At first, we have performed a UV-Vis. titration of 5 μM SRT at pH 3.0 with an increasing concentration of CB7. This titration leads to an isosbestic point at 280 nm indicating the formation of a well-defined complex between SRT and CB7 upon encapsulation (Supplementary Figure 1). However, we did not observe a significant change in the absorption spectra to obtain an accurate binding constant. Thus, we have performed a fluorescence-based titration, which is known to be more sensitive toward complexation, with 10 μM SRT with an increasing concentration of CB7 at pH 3. The fluorescence intensity gradually decreased with increasing concentration of CB7 and saturation could only be achieved after the addition of 1.5 mM CB7 (Figure 3A). Such a decrease of fluorescence intensity indicated the formation of host-guest complex and the supramolecular environment is responsible for the attenuation of the fluorescence intensity. From this titration, we have plotted the fluorescence intensity at 333 nm against the concentration of the CB7, and the data points were fitted using a 1:1 binding equation (see Supplementary Materials). The fitted model provided a binding constant of 23,000 ± 500 M^−1^ (Figure 3B). The binding stoichiometry of the complex was also confirmed experimentally by the Job's plot obtained from NMR titrations (Figure 3C).

The time-resolved anisotropy is also a useful tool to determine the restriction offered by the macrocyclic environment to the encapsulated guest molecule upon binding (Scholtbach et al., 2015; Chandra et al., 2016). If the host-guest complex is formed then the guest would exhibit a non-zero limiting anisotropy decay. Thus, we have measured the anisotropy of free SRT and in the presence of CB7 at pH 3. The anisotropy decay results show that at lower pH (pH~ 3.0) the anisotropy of SRT increases from 0.2 to 0.6 ns upon treatment with CB7 (Figure 3D). The greater anisotropy of SRT in the presence of CB7 at lower pH indicates the hindrance of free rotation caused by the encapsulation of SRT into the hydrophobic cavity. The fluorescence lifetime of SRT did not change upon CB7 encapsulation (Supplementary Figure 2). To understand the binding of deprotonated SRT, we have also performed the binding titration of deprotonated SRT with CB7 at pH 12.0 using fluorescence spectroscopy (Supplementary Figure 3). We have avoided higher pH to reduce the interference from sodium ions from the base. A minute change in the fluorescence intensity indicated the feeble binding with CB7 which is also confirmed by NMR titration (*vide infra*). This goes along the same well-established fact that CB7 binds very weakly with negatively-charged species. Therefore, at higher pH, due to weak complexation, the anisotropy remained unchanged for SRT. The binding titration of SRT with CB7 was also performed in D_2_O at a lower pD. Similar to pH 3.0, we observed a quenching in SRT fluorescence upon complexation with CB7. However, the extent of quenching is lower compared to the same measured in H_2_O (Supplementary Figure 4). The binding constant obtained from these titrations is weakened by a factor of two on moving from H_2_O to D_2_O and this is in accordance with the previous report by Biedermann et al. (2013). Such a reduction in binding strength confirms a strong contribution originated from the host-guest binding enthalpy. Additionally, to understand the SRT and CB7 binding in physiological pH, we have performed fluorescence-based titration (Supplementary Figure 5). Not surprisingly, the binding constant values obtained in H_2_O and D_2_O are similar (Supplementary Figures 5A–C) to that of in lower pH as there is no structural change of the encapsulation SRT molecule.

### NMR-Based Study of SRT•CB7 Complex Formation

After investigating the binding strength of SRT with CB7 using steady-state and time-resolved fluorescence spectroscopy, we were interested to investigate the mode of binding and the structure of the complex in solution. To understand the structure of any host-guest complex in solution and the interaction between them, NMR spectroscopy is one of the best methods.

**Figure 4:**
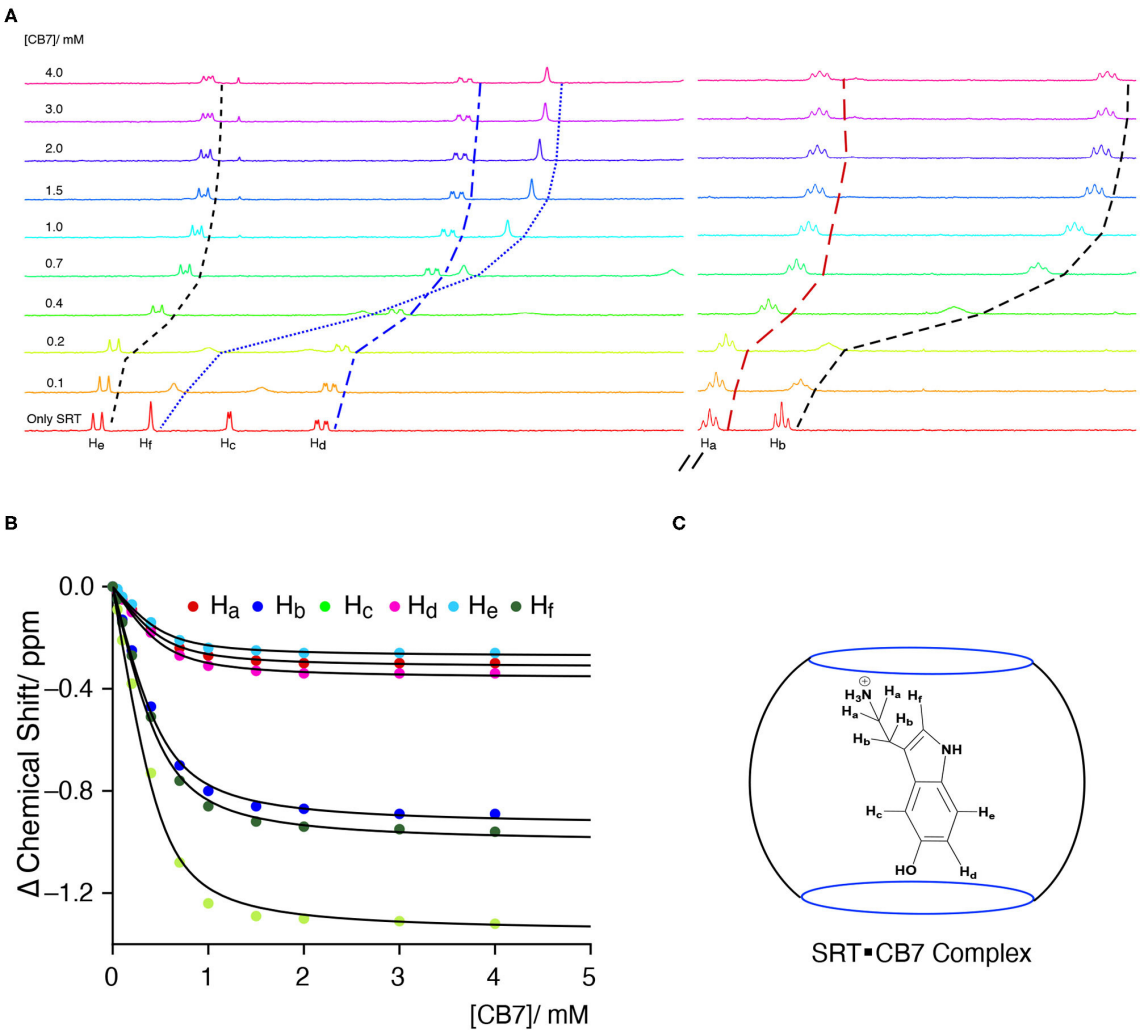
**(A)** NMR-based titration of 0.5 mM SRT with increasing concentration of CB7 up to 4.0 mM at pD 2.8; left part showing the aromatic region and right part the aliphatic region of NMR titration, **(B)** fitted plot of the change in Complexation Induced Shift (CIS) vs. concentration of CB7, **(C)** schematic representation of plausible SRTH^+^•CB7 complex.

The Complexation Induced Shift (CIS) in the NMR chemical shift due to the re-location of the guest molecule in the macrocyclic host cavity can allow us to understand the nature of the interaction and geometry of the complex (Hunter and Packer, 1999; Gomila et al., 2002). The CIS of the guest signal is monitored to quantify the binding strength of complexation for a host-guest system. The ^1^H NMR signal showing an up-field CIS value indicates an encapsulation of the guest molecules in the hydrophobic environment while downfield signals indicate the positioning of the hydrogen just in the interface of the host cavity. We have performed an NMR titration of 0.5 mM SRT with an increasing concentration of CB7 up to 4.0 mM at two different pH. Each proton present in the SRT was assigned before analyzing the titration data (Figure 4A). At lower pH, *ca*. pD 3.0, a strong up-field CIS was observed in H_b_, H_c_, and H_f_ which indicate that those hydrogen atoms of SRT are present in the core of the CB7 cavity (Figure 4C). On the other hand, a small up field CIS H_a_, H_d_, and H_e_ suggested encapsulation of these protons are present relatively nearer to the portal of the CB7. The full-length NMR spectra are shown in Supplementary Figure 6. To evaluate the binding strength between SRT and CB7, we plotted the change in the chemical shift of six different protons present in SRT against the concentration of CB7 added during the NMR titration (Figure 4B). The data points were fitted by 1:1 binding equation and get the average binding constant (9,200 ± 1,200) M^−1^ which is very similar to the value obtained from fluorescence binding titration in D_2_O. Further, we have also performed an NMR titration at pD 7.4 with 0.2 mM SRT and an increasing concentration of CB7 up to 3.2 mM. The average binding constant value obtained using the 1:1 equation from fitting all the NMR peaks at pD 7.4 is (11,500± 1,800) M^−1^ (see Supplementary Figures 7, 8). This is similar to the value measured at pD 7.4 using fluorescence spectroscopy. Such a binding geometry could eventually cause a significant shift in the p*K*_a_ value. Unfortunately, we could not monitor the p*K*_a_ value of SRT in SRT•CB7 complex as the p*K*_a_ value was out of range in experimental pH conditions. Nonetheless, it is interesting to note that at pH 13 there is no change in CIS for host-guest complexation (Supplementary Figure 9). Hence, it is evident that no complex formation occurs in basic pH and the shift in the p*K*_a_ is less than two logarithmic units owing to the competition provided by the sodium ions present in the basic pH range.

### Understanding the Role of Cs^+^ as a Competitor on the Stability of SRT•CB7

**Figure 5:**
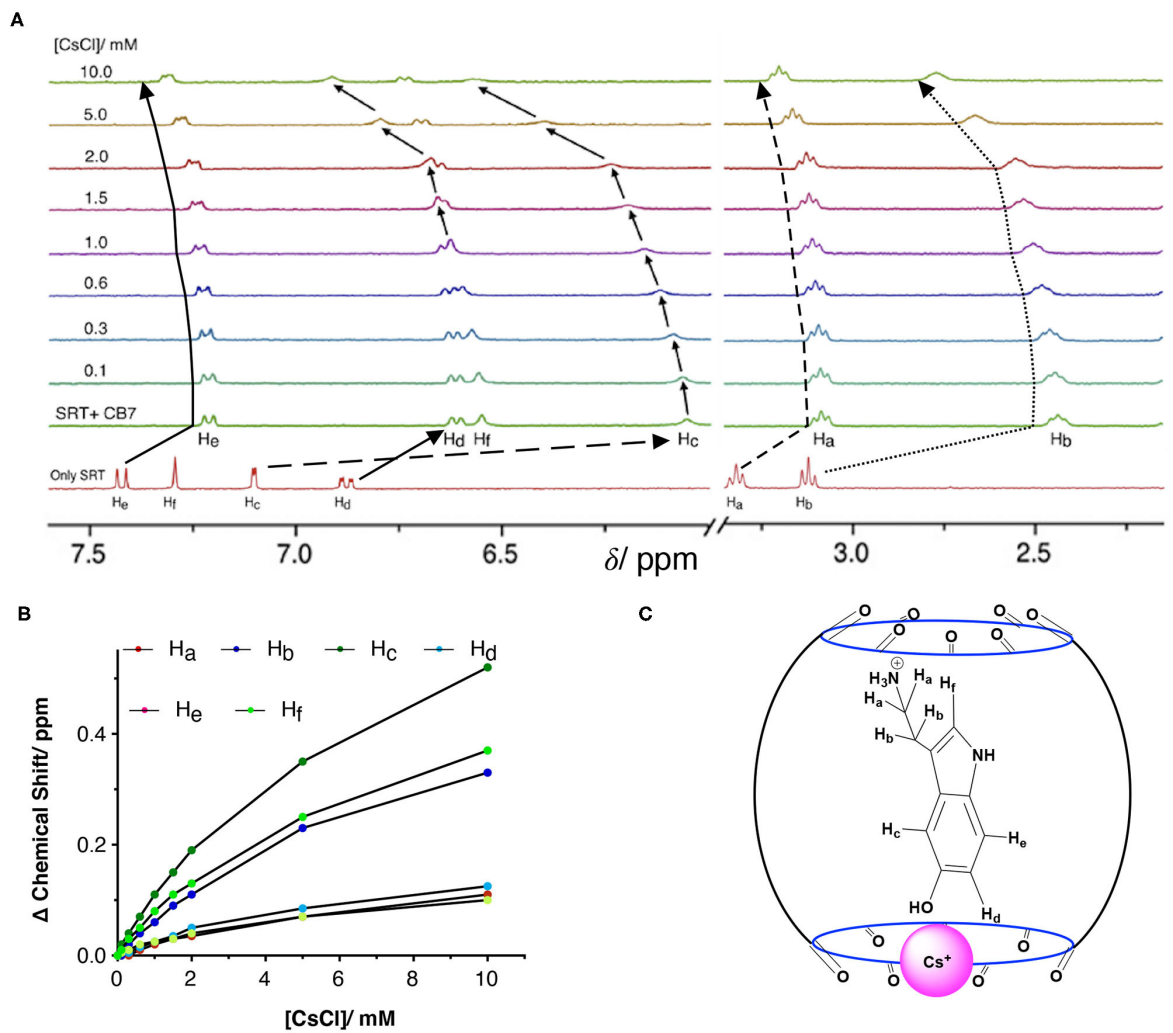
**(A)** NMR-based titration of the SRT•CB7 (0.5 mM SRT and complex with increasing concentration of CsCl up to 10 mM at pD 2.5; part showing the aromatic region and right part the aliphatic region of NMR spectra. **(B)** A fitted plot of the difference in chemical shift (in ppm) against the concentration of CsCl. **(C)** Schematic representation of SRT•CB7•Cs^+^ complex.

To understand the stability, stoichiometry, and the depth of inclusion of the SRT•CB7 complex, we have performed a competition titration experiment with the help of a bulky mono-cationic alkali metal. The selection of cesium ion was based on mainly two reasons. Firstly, it can act as the lid on the top/bottom of the CB7 portal due to the ion-dipole interaction. As the previous studies show that Cs^+^ has a strong affinity to form a 1:1 complex with CB7 (Whang et al., 1998; Pichierri, 2013). Secondly, it is an NMR innocent alkali metal ion. Hence, we carried out the NMR-based competition assay at pD 2.8 of the pre-formed SRT•CB7 complex with an increasing concentration of CsCl up to 10 mM (Figure 5A, Supplementary Figure 10). In this experiment, we anticipated the displacement of bound SRT by Cs^+^. With the increasing concentration of CsCl, the ^1^H NMR signal showed a small downfield shift in the peak positions. To our surprise, the extent of the downfield shift strongly indicated no displacement of SRT from the CB7 cavity (Figure 5B). The Cs^+^ ion binds to the portal of the CB7, and with increasing the concentration it re-orientates SRT inside the CB7 cavity. This was further validated with a fluorescence titration by adding CsCl in a pre-formed SRT•CB7 complex (see Supplementary Figure 11). In fact, the initially up-field shifted peaks due to SRT•CB7 complexation were partially shifted to down-field. Hence, we did not observe a full recovery of the peak position. This confirms that there is a formation of a ternary complex formation (SRT•CB7•Cs^+^, Figure 5C) where Cs^+^ ion acts as a lid possibly from the one side of the portal.

### Molecular Docking Studies

**Figure 6:**
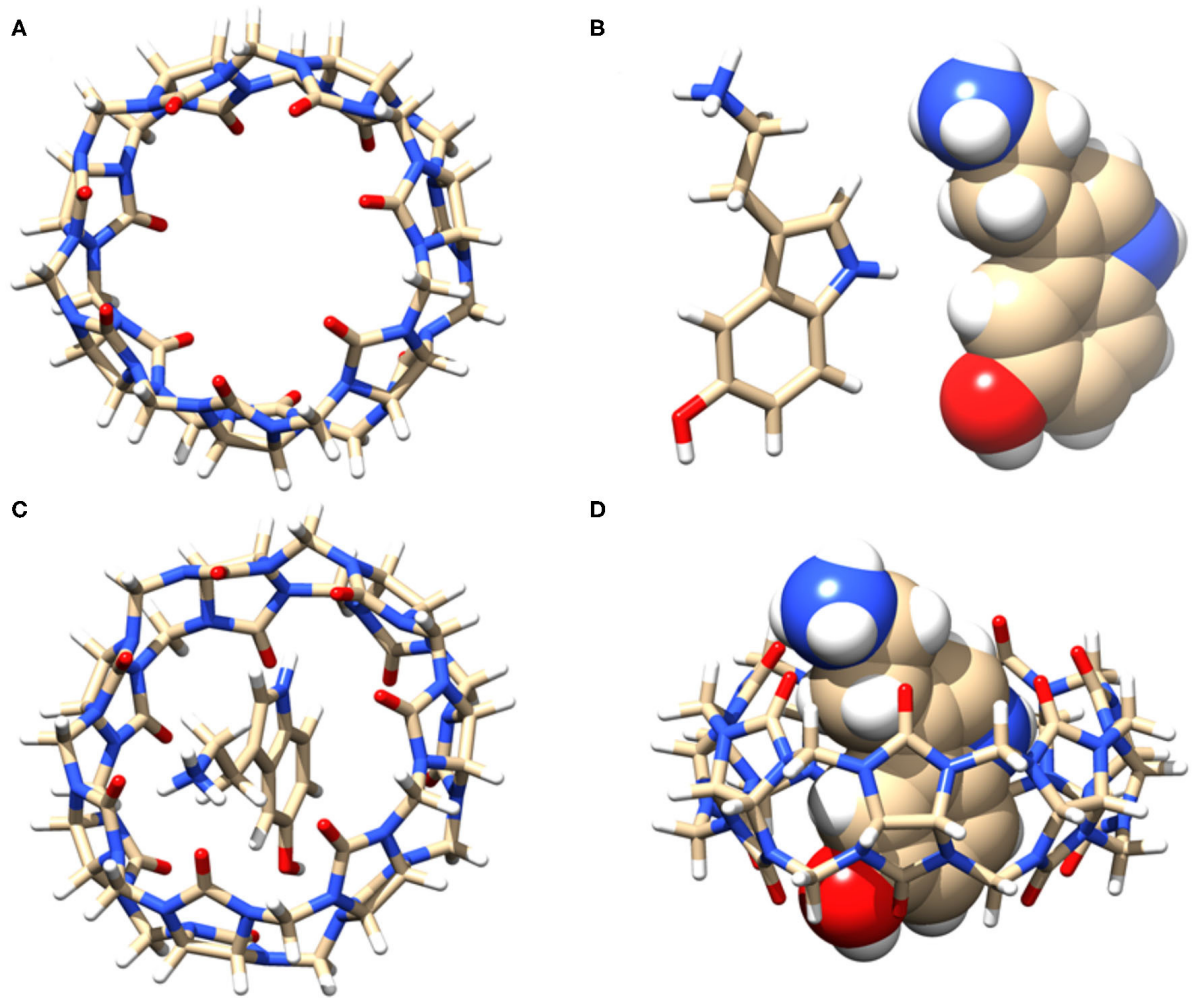
**(A)** Stick model of the guest CB7 (top view), **(B)** stick and sphere model of SRT in acidic pH, **(C)** top view, and **(D)** side view of the of 1:1 inclusion complex of SRT•CB7 and **(D)**. The carbon, hydrogen, nitrogen, and oxygen atoms are shown in gray, white, blue, and red, respectively.

Based on the NMR titration experiments, it is evident that SRT resides inside the cavity of CB7 while the amine group protrudes along with the portal. Even as complete inclusion of the main framework was suggested by the proton chemical shifts, the actual orientation could not be understood. Hence, we performed molecular docking using the PatchDock server (Duhovny et al., 2002; Schneidman-Duhovny et al., 2005). The 3D structure of CB7 was obtained from the PDBe database and optimized by the PM3 method using Gaussian 09W. The protonated structure of SRT was optimized using the B3LYP method at 6-31G^*^ level. Several probable structures were received from the PatchDock server program based on the geometric shape complementarity score, approximate interface area of the complex and, atomic contact energy (ACE). The structure of the individual molecules and inclusion complex, shown in Figures 6A–D, has the highest geometric shape complementarity score 3,056, an approximate interface area of 319.90 Å^2^, and ACE is −349.13 kcal/mol (Supplementary Table 1). The orientation of SRT inside CB7 well corroborates with the proton chemical shifts obtained from the NMR titration.

## Conclusions

In summary, we have extensively explored the encapsulation of a well-known neurotransmitter molecule serotonin with a rigid water-soluble macrocyclic host CB7 using different spectroscopic techniques and molecular docking. Primarily, the pH-dependent photophysical properties of serotonin were investigated. The host-guest complexation between CB7 and serotonin in different pH revealed an efficient binding of the amine-protonated species of serotonin, whereas, in basic pH, no complex was formed. The 1:1 stoichiometry of the complex was assessed using Job's plot. Interestingly, we observed a binding constant difference of two times in favor of H_2_O compared to D_2_O due to strong enthalpic contribution in the complexation between serotonin and CB7. Further confirmation of the complexation was obtained from fluorescence anisotropy and NMR. From NMR titration, the complexation induced chemical shift values were evaluated to understand the solution structure of the complex. Subsequently, Cs^+^ driven competition of bound serotonin indicated the formation of the ternary complex originated from the pre-formed 1:1 complex where Cs^+^ acts as the lid. Finally, the molecular docking studies were performed to validate the geometrical orientation and stoichiometry of the complex. We believe this study will be useful for serotonergic drug delivery and treatment of serotonin syndrome, considering the numerous roles of serotonin in physiological and pathophysiological processes and being one of the most important neurotransmitters.

## Data Availability Statement

All datasets generated for this study are included in the article/Supplementary Material.

## Author Contributions

AK designed the project. FC and TD performed all the experiments and calculations. They have plotted and analyzed the data with the help of AK. All authors contributed to manuscript writing.

## Conflict of Interest

The authors declare that the research was conducted in the absence of any commercial or financial relationships that could be construed as a potential conflict of interest.
