# Supplementary material for: Supramolecular Encapsulation of a Neurotransmitter Serotonin by Cucurbit[7]uril
Source: Front Chem. 2020 Oct 23;8:582757. doi: 10.3389/fchem.2020.582757 (PMC7645158; doi:10.3389/fchem.2020.582757)
Supplement: Supplementary file 1 [file Data_Sheet_1.PDF]

Supplementary Material

Supramolecular Encapsulation of Serotonin Neurotransmitter by Cucurbit[7]uril

Falguni Chandra,[1#] Tanoy Dutta,[1#] Apurba L. Koner[1]*

[1]Bionanotechnology Laboratory, Indian Institute of Science Education and Research Bhopal, Department of Chemistry, Bhopal Bypass Road, Bhauri, Bhopal, Madhya Pradesh 462 066, India

* Corresponding author: Apurba L. Koner, akoner@iiserb.ac.in, [#]these authors contributed equally to this work

1:1 Binding equation used for fitting:

Fluorescence intensity= $I_{SRT\bullet CB7} + (I_{SRT} - I_{SRT\bullet CB7})\times\frac{[SRT]}{[SRT]_0}$, where $I_{SRT\bullet CB7}$=intensity of the complex, I_{SRT}=intensity of only SRT without CB7, $[SRT] = ([SRT]_0 - [CB7]_0 - {}^{1}/_{K})/2 + \sqrt{(\left([SRT]_0 + [CB7]_0 + {}^{1}/_{K}\right)^2 - 4[SRT]_0[CB7]_0)}$), K= Binding constant.

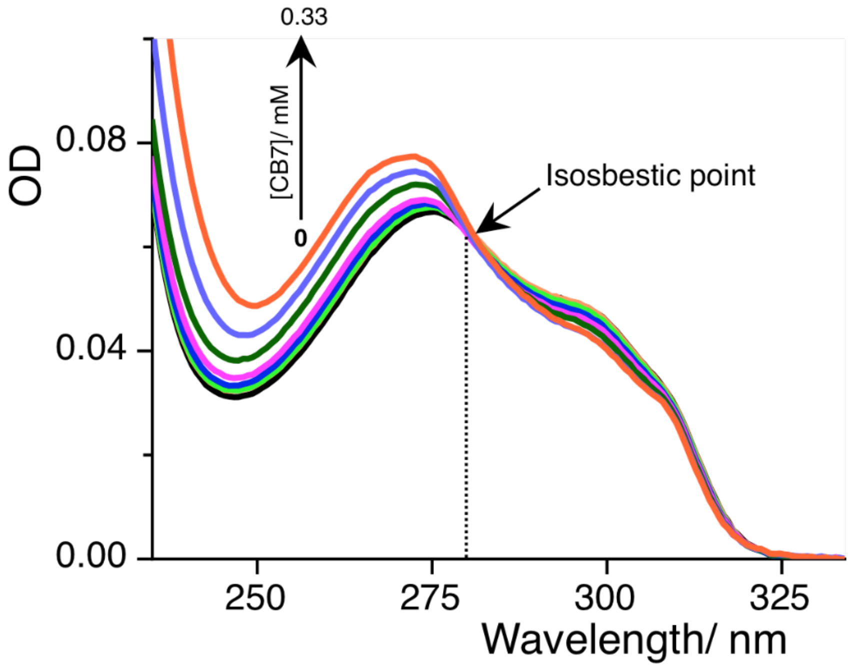

Figure S1: UV-Vis. spectra of 5 μM SRT with increasing concentration of CB7 at pH 3

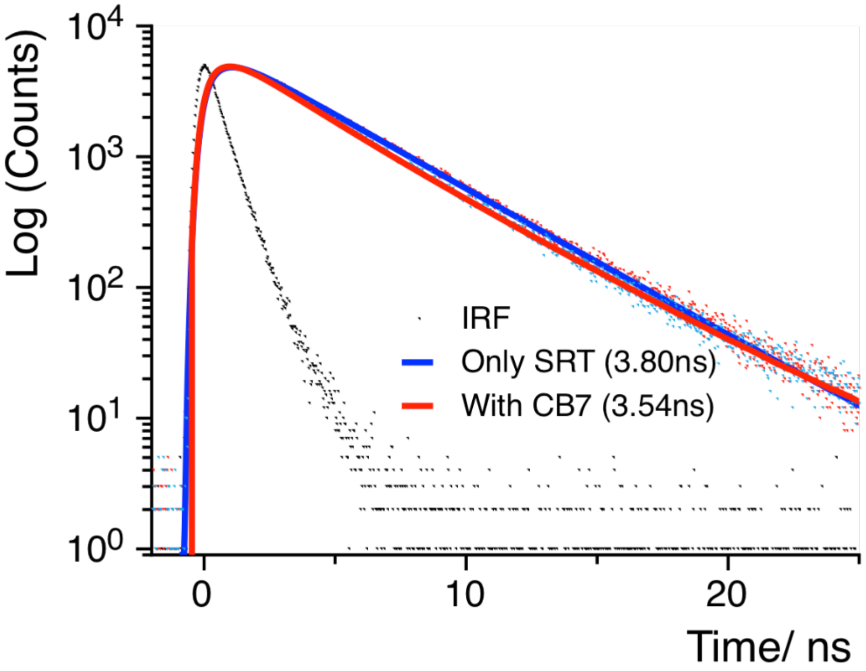

Figure S2: Fluorescence lifetime decay of SRT without and with CB7 at pH 3

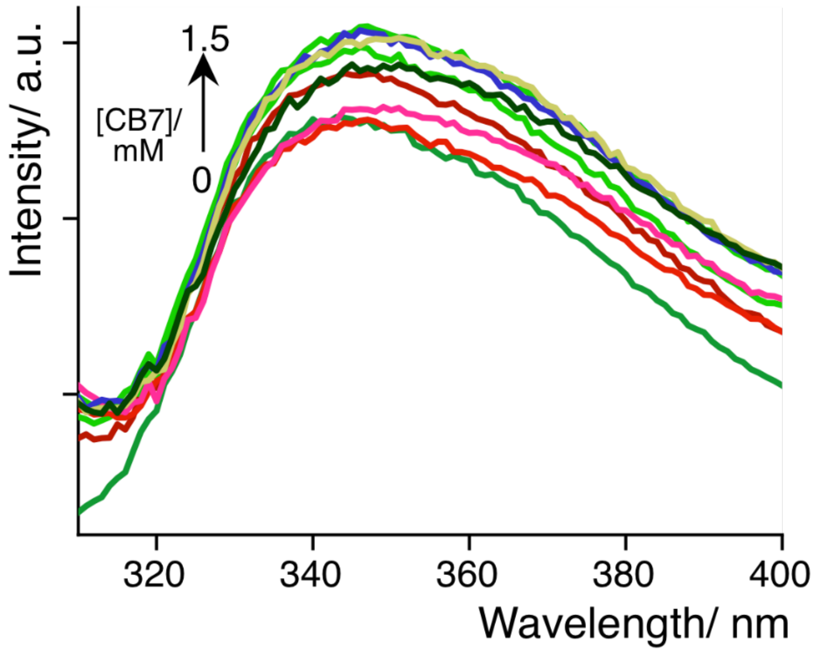

Figure S3: Fluorescence spectra of 5 μM SRT with increasing concentration of CB7 at pH 12

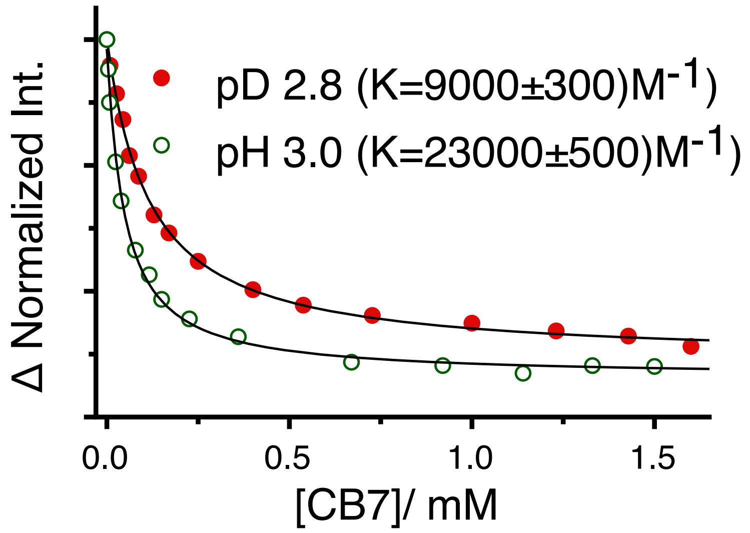

Figure S4: Effect of heavy water on the binding constant of protonated SRT with CB7 at pH/pD ~3. The binding constant in D_2O is *ca*. 0.4 times lower compared to the same in H_2O.

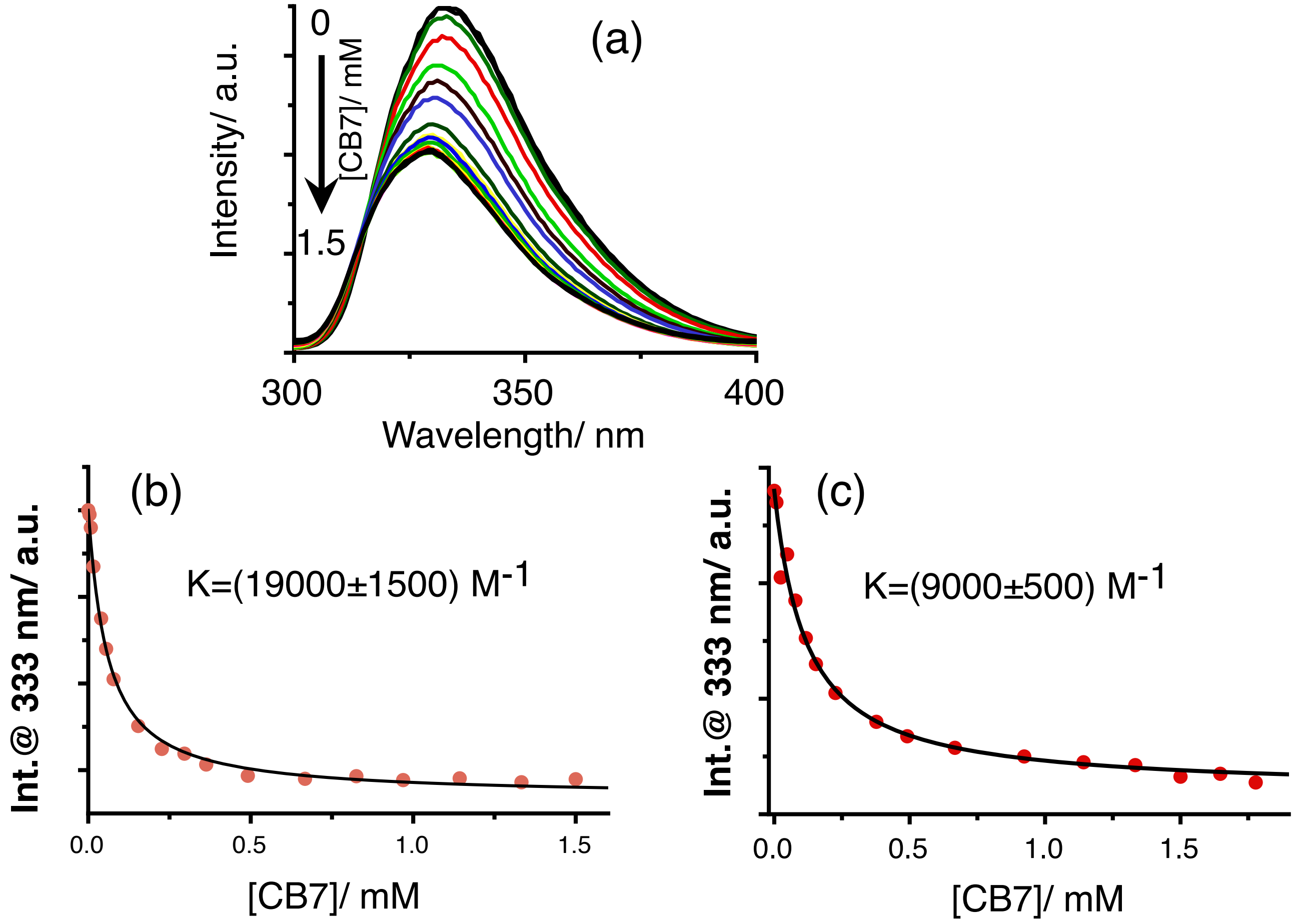

Figure S5: Binding studies of SRT with CB7 in physiological pH. (a) Fluorescence titration of 10 μM SRT with CB7 at pH 7.0, (b) plot of the fluorescence intensity at 333 nm measured at pH 7.0 with increasing concentration of CB7; solid line shows the 1:1 fitted function, and (c) plot of the fluorescence intensity at 333 nm measured at pD 7.4 with increasing concentration of CB7; solid line shows the 1:1 fitted function.

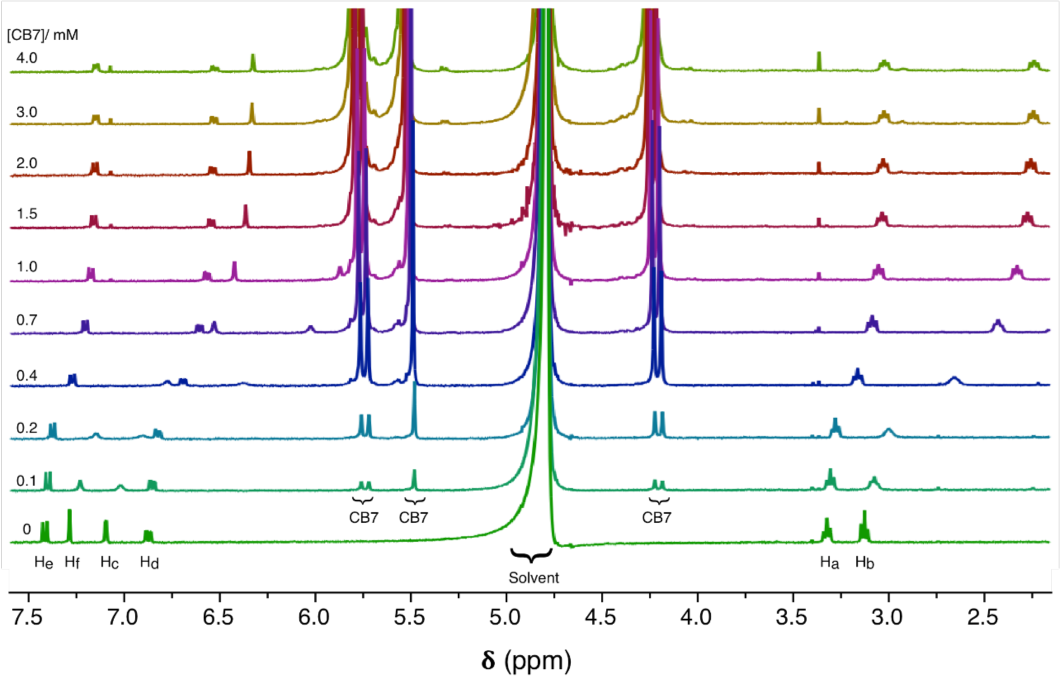

Figure S6: Full-length NMR based titration of 0.5 mM SRT with increasing concentration of CB7 up to 4 mM at pD 2.5

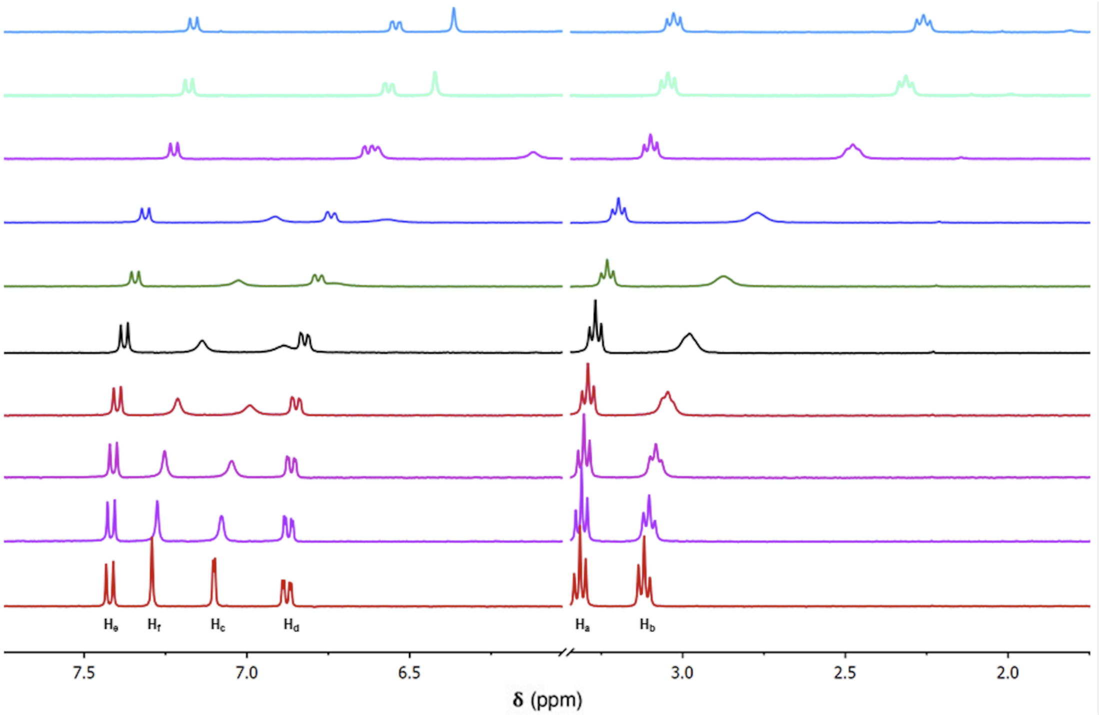

Figure S7: Full-length NMR based titration of 0.5 mM SRT with increasing concentration of CB7 up to 4 mM at pD 7.4

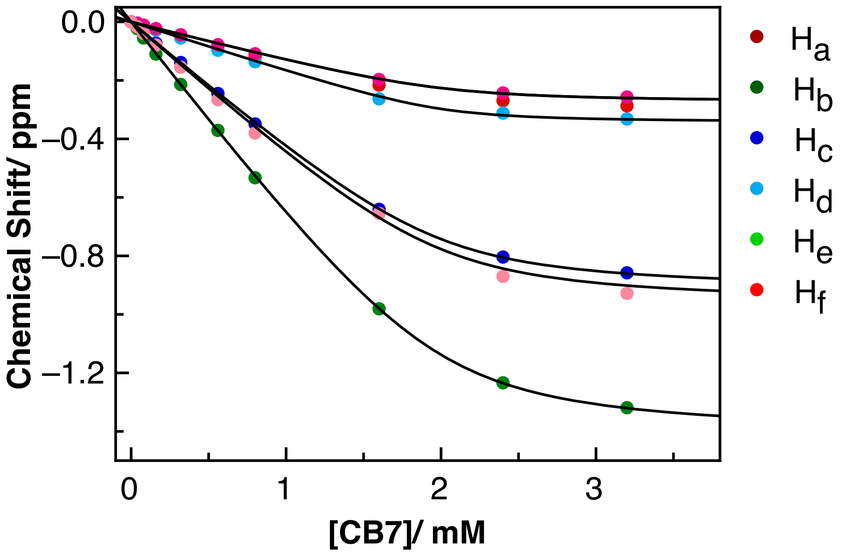

Figure S8: Full-length NMR based titration of 0.5 mM SRT with increasing concentration of CB7 up to 4 mM at pD 7.4

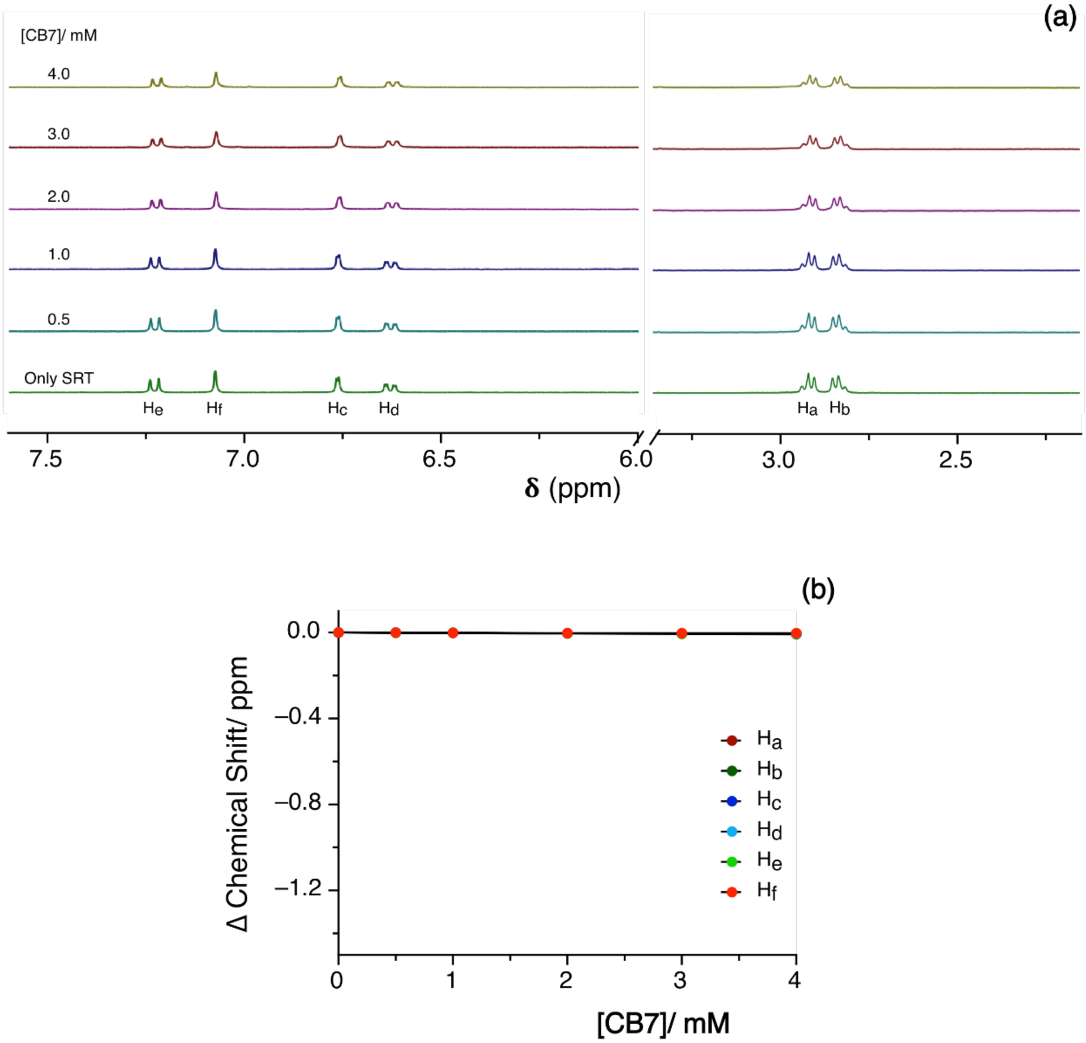

Figure S9: (a) NMR-based titration of 0.5 mM SRT with increasing concentration of CB7 up to 4 mM at pD 13; left part showing the aromatic region and right part the aliphatic region of NMR spectra. (b) A plot showing the difference in chemical shift (ppm) vs. concentration of CB7.

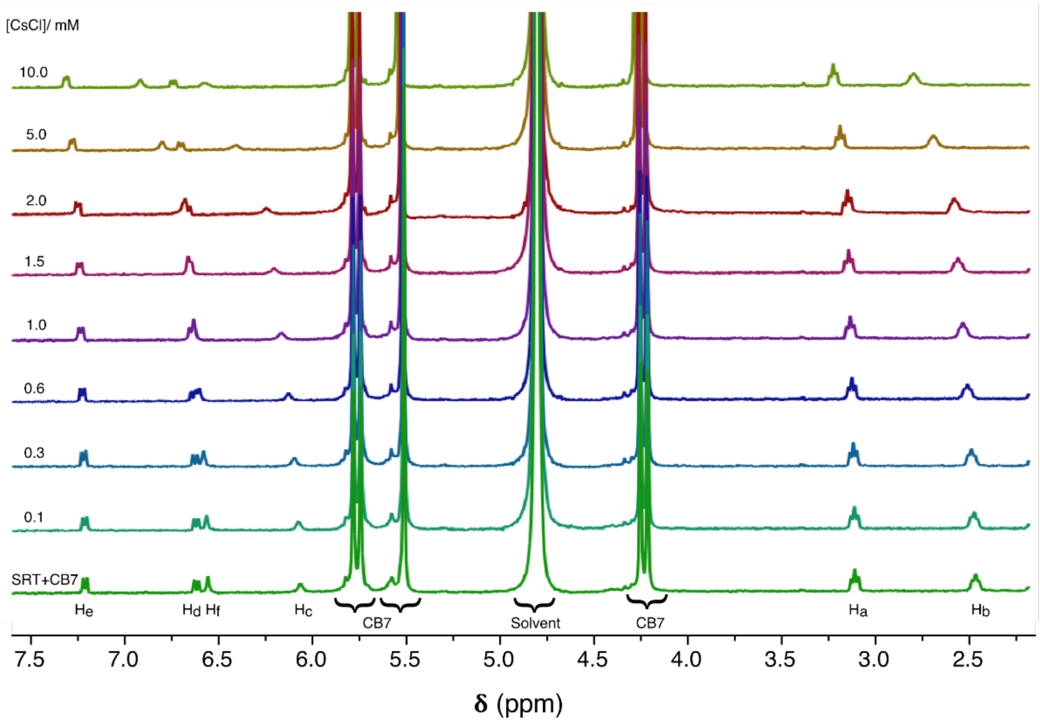

Figure S10: Full-length NMR-based titration of SRT•CB7 complex with increasing concentration of CsCl upto10 mM at pD 2.5

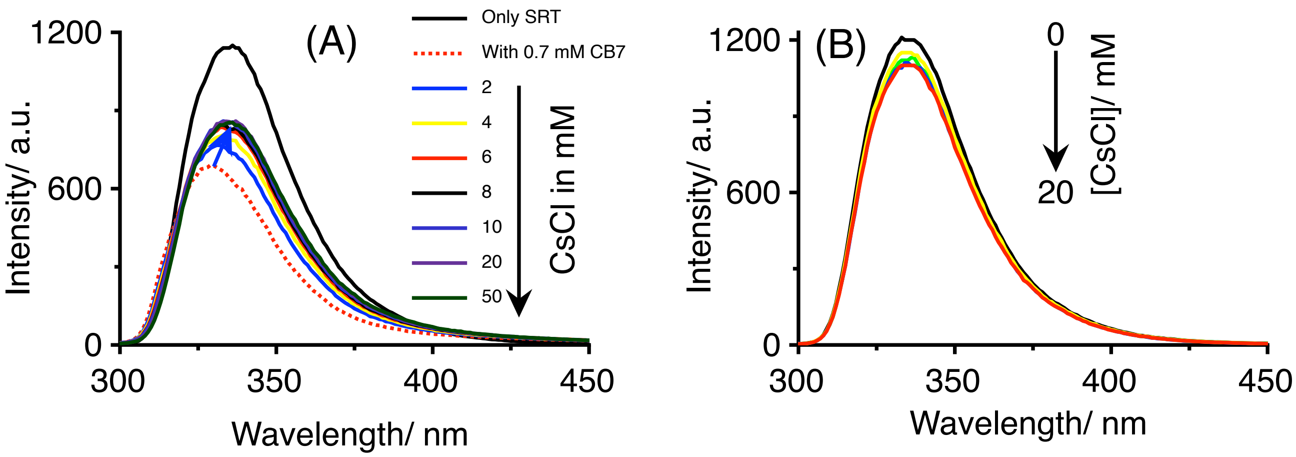

Figure S11: Understanding the effect of CsCl on the emission properties of SRT and SRT•CB7 complex (a) Fluorescence titration with CsCl using a pre-formed SRT•CB7 complex (10 µM SRT and 0.7 mM CB7) at pH 3.0; (b) Effect of CsCl on the emission of SRT at pH 3.0.

Table S1: Docking results of the top 10 docked models of SRT•CB7 inclusion complex

Serial No.	Geometric shape complementarity score	Approximate interface area of the complex, Å^2	Atomic contact energy (ACE), kcal/mol
1	3056	319.90	-349.13
2	2998	311.30	-324.34
3	2688	347.60	-339.50
4	2668	337.70	-324.45
5	2568	330.60	-290.33
6	2504	344.10	-327.25
7	2490	333.50	-316.24
8	2410	279.50	-275.56
9	2382	304.40	-298.24
10	2266	316.00	-292.69
