# Supplementary figures and images for: Supramolecular Encapsulation of a Neurotransmitter Serotonin by Cucurbit[7]uril
Source: Front Chem. 2020 Oct 23;8:582757. doi: 10.3389/fchem.2020.582757 (PMC7645158; doi:10.3389/fchem.2020.582757)

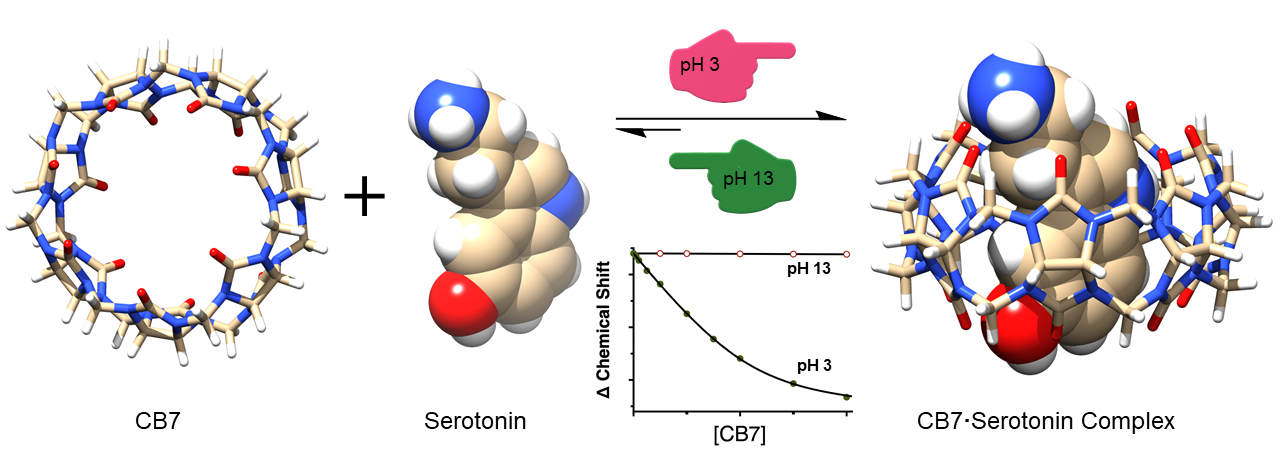

Supplement: Supplementary file 2 [file Image_1.TIF]
